# Validity and Reliability of the Greek Version of Pittsburgh Sleep Quality Index in Chronic Non-Specific Low Back Pain Patients

**DOI:** 10.3390/healthcare12050557

**Published:** 2024-02-28

**Authors:** Kyriakos Petropoulakos, Vasiliki Papakonstantinou, Smaragda Pentsi, Eftychia Souzou, Zacharias Dimitriadis, Evdokia Billis, Georgios Koumantakis, Ioannis Poulis, Savvas Spanos

**Affiliations:** 1 Human Performance and Rehabilitation Research Laboratory, School of Health Sciences, University of Thessaly, 35132 Lamia, Greeceipoulis@uth.gr (I.P.);; 2 Health Assessment and Quality of Life Research Laboratory, University of Thessaly, 35132 Lamia, Greece; 3 Faculty of Physiotherapy, School of Health Rehabilitation Sciences, University of Patras, 26504 Patra, Greece; 4 Research Laboratory of Advanced Physiotherapy, School of Health & Care Sciences, University of West Attica, 12241 Athens, Greece; gkoumantakis@uniwa.gr

**Keywords:** sleep disturbance, chronic non-specific low back pain, Pittsburgh sleep quality index, validity, reliability

## Abstract

The purpose of this study was to investigate psychometric properties of the Greek translation of Pittsburgh Sleep Quality Index (GR-PSQI) in a Greek chronic non-specific low back pain (CNSLBP) sample, thus, providing insight on its clarity and acceptability as a widely used sleep assessment tool in clinical practice. Asymptomatic volunteers (n = 73) and CNSLBP volunteers (n = 47), participated in the study. For the assessment of construct validity, the known-groups method was used. Thus, all the participants (asymptomatic and CNSLBP) completed the GR-PSQI. For the assessment of concurrent validity, the CNSLBP participants additionally completed the following validated questionnaires for depression, insomnia and sleep quality: Beck Depression Inventory Questionnaire (BDI), Insomnia Severity Index (ISI), and Sleep Quality Numeric Rating Scale (SQNRS). For the assessment of test–retest reliability, the CNSLBP participants completed the GR-PSQI a second time, one week after the first time. The results showed excellent test–retest reliability (ICC = 0.969, SEM = 0.90, SDD = 2.49%) and internal consistency (Cronbach *α* = 0.985), moderate to good concurrent validity (from *r* = 0.556 to *r* = 0.860) among PSQI, BDI, SQNRS, and ISI, as well as excellent construct validity (*p* = 0.000) between the two groups. The Greek translation of PSQI could be a valuable tool for Greek healthcare professionals in both clinical and research environments.

## 1. Introduction

Sleep is a daily living activity, thus, good sleep quality is crucial for overall well-being. It impacts mood, cognitive function, immune system and even physical health. It is reported that poor sleep can lead to various health issues and diminish the overall quality of life [1].

Sleep quality could be unfavorably altered by a plethora of pathological conditions. It is usual to meet people affecting difficulties in falling asleep, in sleeping without disturbances during the night, and in waking up early in the morning [2]. As a result, these people face symptoms such as sleepiness, fatigue, attentional disorders, bad mood, and cognitive difficulties, during the day [3,4,5,6,7]. Additionally, sleep disorders may be one of the reasons for obesity and depression [2]. Sleep assessment is essential in rehabilitation since it constitutes a contributing parameter in patients’ functional status [3,8].

Chronic non-specific low back pain (CNSLBP) is the most frequent cause of disability for middle-aged people [9]. Guidelines for CNSLBP often recommend the early recognition of psychosocial factors, such as depression, that could influence patients’ recovery and contribute to poor outcomes [10]. Additionally, CNSLBP influences different parts of the daily living such as sleep quality [11,12]. More than 50% of people facing CNSLBP deal with sleep disturbances [13]. It has been found that people facing sleep disturbances often develop chronic pain and vice versa [2,13]. It is proposed that rehabilitation programs should emphasize sleep quality apart from the other targets, to provide better control of this condition. Since sleep assessment is essential in rehabilitation of CNSLBP patients, there is the need for valid, reliable, as well as easy to use in routine clinical practice, sleep assessment tools.

The Pittsburgh Sleep Quality Index (PSQI), originally developed in English, is a concise self-administered questionnaire consisting of 19 items. It is designed to evaluate the subjective aspects of sleep, including both the quality and quantity of sleep, as well as sleep habits and the frequency of sleep disturbances in adults. This assessment covers a period of one month. The PSQI is user-friendly and can be conveniently completed in about 5 min. It has been evaluated for its validity (acceptable), test–retest reliability (*r* = 0.85 *p* < 0.001 between two occasions), internal consistency (Cronbach *α* = 0.83) and it is the most frequently used questionnaire for assessing sleep quality. A cut-off >5 showed 89.6% sensitivity and 86.5% specificity [14].

PSQI has been translated in 23 languages and has been widely selected for various studies due to its concise, straightforward, valid, reliable, and user friendly nature (Table 1).

Kotronoulas et al. [15] translated PSQI into Greek language and assessed its psychometric properties in patients with cancer during active-phase of chemotherapy treatment. They discovered high test–retest reliability (ICC = 0.82) and acceptable internal consistency (Chronbach’s *α* = 0.76).

However, patients with cancer present different characteristics and as a result different reasons for sleep disturbances from that of CNSLBP. Sleep problems due to stress, anxiety and depression, may be manifested during all phases of the cancer experience and in direct relation to cancer diagnosis or to poor prognosis [15]. Difficulty sleeping may follow stressful life events, such as a cancer diagnosis, that produce worry or concern regarding the disease, treatment, or impact on family members [38]. Additionally, many of the pharmacologic agents that cancer patients receive are associated with changes in sleep architecture [39]. On the other hand, an increase in self-report pain intensity and the depression that follows is directly related to a reduction in self-report sleep quality in CNSLBP patients [13,40]. Therefore, there is additional need for the assessment of the psychometric properties of GR-PSQI in CNSLBP patients before its use on such population.

## 2. Materials and Methods

The purpose of the current study was to investigate psychometric properties (construct and concurrent validity, internal consistency as well as test–retest reliability) of the PSQI-GR in a Greek CNSLBP sample, thus, providing insight on its clarity and acceptability as a widely used sleep assessment tool in clinical practice.

### 2.1. Design and Participants

A sample size calculation was performed for the main purposes of our study. For the correlation between the PSQI-GR and other relative instruments in CNSLBP individuals, sample size calculation was performed with G*Power 3.1.9.4 and revealed that for the examination of this correlation (two tailed hypothesis, *α* = 0.05, power 80%, rH1 = 0.5), a sample size of at least 29 participants was needed. For test–retest reliability in CNSLBP individuals, sample size calculation (ρ_0_ = 0, ρ_1_ = 0.6, *α* = 0.05, power = 80%, k = 2), revealed that at least 18 participants were required [41].

A prospective, consecutive sampling approach was followed for the present study.

Thus, 120 people were screened for eligibility. All were identified as eligible. In total, 73 asymptomatic volunteers (36 males and 37 females, age range 19–61 years) and 47 volunteers (22 males, 25 females, age range 18–62 years) suffering from chronic non-specific low back pain (CNSLBP), participated in this study in order to increase statistical power.

Asymptomatic participants were recruited from the students and staff of our university. The inclusion criteria were: people between 18 and 70 years old, without any pain, Greek native speakers, Greek inhabitants, presenting good cognitive function. The exclusion criteria were: people with sleep disturbances due to pathological disorder diagnosed by a physician, pregnancy, addicted to drugs or alcohol, suffering from bipolar or psychiatric disease, neurological disorders, and current musculoskeletal injuries or operation.

CNSLBP participants were recruited from the outpatient departments of hospitals as well as from consulting rooms. The inclusion criteria were: people between 18 and 70 years old, with non-specific low back pain lasting for more than 3 months and with intensity above 3/10 according to Numeric Rating Scale (NRS), with or without radiation, who did not receive operation as well as any sleep medication the last 12 months, Greek native speakers, Greek inhabitants, presenting good cognitive function. The exclusion criteria were: people with sleep disturbances due to pathological disorder diagnosed by a doctor, red flags (fever, genitourinary symptoms, spinal injection, vertebral fracture, malignancy) [42], pregnancy, addiction to drugs or alcohol, suffering from bipolar or psychiatric disease, neurological disorders and musculoskeletal injuries or operation [43,44].

### 2.2. Procedure

A signed informed consent was obtained from all participants (47 CNSLBP and 73 asymptomatic) before their enrolment in the study. The study protocol was carried out in accordance with the Code of Ethics of the World Medical Association (Declaration of Helsinki) and approved by the Research Ethics Committee of our Faculty (500SE2/2 March 2021).

Demographic and anthropometric data such as age, sex, height, and weight were recorded.

For the assessment of construct validity, the known-groups method was used. Thus, all the participants (47 CNSLBP and 73 asymptomatic) completed the GR-PSQI. Construct validity was assessed by comparing the results of asymptomatic participants with those suffering from CNSLBP since asymptomatic people present much lower percentages of sleep disturbances [13,38].

For the assessment of concurrent validity, the CNSLBP participants additionally completed the Greek version of Beck Depression Inventory Questionnaire (BDI), Insomnia Severity Index (ISI), and Sleep Quality Numeric Rating Scale (SQNRS). Concurrent validity of the PSQI-GR was assessed by correlating the results of the PSQI-GR with the results of the above questionnaires (BDI, ISI, SQNRS).

For the assessment of test–retest reliability, the CNSLBP participants completed the GR-PSQI a second time, one week after the first time. This time interval was agreed by the authors as appropriate for both avoiding memory bias and potential changes of the participants’ condition.

#### 2.2.1. Pittsburgh Sleep Quality Index (PSQI)

The questionnaire is a self-administrated tool consisting of 19 questions, designed to evaluate various aspects of sleep in adults. This assessment covers a period of one month and touches on aspects such as the quality and quantity of sleep, sleep habits, and disturbances during sleep. The questionnaire is divided into seven areas: subjective sleep quality (1 question), time taken to fall asleep or sleep latency (2 questions), total sleep duration (1 question), regularity and efficiency of sleep (3 questions), frequency of sleep disturbances (9 questions), reliance on sleep medications (1 question), and the impact of sleep on daytime functioning (2 questions). Each of these sections is scored on a scale from 0 to 3, with 0 representing no issues and 3 indicating severe difficulties. The scores from these seven sections are combined to form an overall score that reflects the individual’s global sleep quality. This total score can range from 0 to 21, with higher scores signifying more sleep problems and hence poorer sleep quality. A global score of 5 or more typically suggests poor sleep quality, effectively distinguishing between those with good and poor sleep. The questionnaire is user-friendly and quick to complete, typically taking about 5 min [14].

#### 2.2.2. Beck Depression Inventory Questionnaire (BDI)

The Beck Depression Inventory (BDI) was developed based on observations of common attitudes and symptoms prevalent among depressed psychiatric patients, and less so among non-depressed ones. These observations were methodically transformed into a list of 21 distinct symptoms and attitudes. Each item on this list can be rated on a scale from 0 to 3, reflecting the severity of the symptom. Importantly, the selection of these items was based on their relevance to measuring the severity of depression, rather than aligning with any specific theoretical framework of depression. The 21 items encompass a range of symptoms and attitudes including: (1) Mood; (2) Pessimism; (3) Feeling of Failure; (4) Dissatisfaction; (5) Guilt; (6) Feeling Punished; (7) Dislike of Self; (8) Self-blame; (9) Thoughts of Suicide; (10) Tendency to Cry; (11) Irritability; (12) Withdrawal from Social Interaction; (13) Indecision; (14) Altered Body Image Perception; (15) Hindered Work Performance; (16) Sleep Problems; (17) Fatigue; (18) Decreased Appetite; (19) Weight Loss; (20) Preoccupation with Physical Symptoms; and (21) Reduced Sexual Interest. Although the BDI was initially designed to be administered by trained interviewers, it is most often self-administered. When self-administered, the instrument generally takes 5–10 min to complete and is scored by summing the ratings given to each of the 21 items [45]. The Center for Cognitive Therapy has distributed the following guidelines for BDI cut-off scores with patients diagnosed as having an affective disorder: none or minimal depression is <10; mild to moderate depression is 10–18; moderate to severe depression is 19–29; and severe depression is 30–63 [46]. It has been assessed for its validity and reliability in people with Parkinson’s disease [47], in adolescent psychiatric inpatients [48], in family caregivers of children with chronic diseases [49], as well as in people with multiple sclerosis [50].

#### 2.2.3. Insomnia Severity Index (ISI)

The Insomnia Severity Index (ISI) is a tool comprising seven questions, aimed at offering a comprehensive measure of Insomnia’s severity. Each question is evaluating a Likert scale ranging from 0 to 4. For the purpose of effectively identifying sleep issues, a score of 8 or above is considered to have the best balance of sensitivity and specificity. The ISI is recognized as both reliable and valid for assessing insomnia, particularly in cases of primary insomnia [51].

#### 2.2.4. Sleep Quality Numeric Rating Scale (SQNRS)

The Sleep Quality Numeric Rating Scale (SQNRS) is a method used to evaluate a person’s perceived quality of sleep over the previous week. This is performed by making a mark on a 10 cm line. On this scale, the far left represents the lowest possible sleep quality, scored as 0, while the far right indicates the highest quality of sleep, scored as 10. The SQNRS has been widely implemented in past research for detecting different emotions or feelings and has been established as both reliable and valid [15].

### 2.3. Statistical Analysis

The collected data were processed using the Statistical Package for the Social Sciences software (SPSS version 22.0, IBM Corporation, Armonk, NY, USA). A preliminary descriptive analysis and the Kolmogorov–Smirnov test for normality were used to ascertain that the data for any of the variables were normally distributed. For test–retest reliability, the model 2.1 (two-way random, absolute agreement, single measures) Intraclass Correlation Coefficient (ICC) was estimated and their 95% confidence interval (CI). Internal consistency was examined with the Cronbach’s *α*. An ICC (model 2, k) with coefficients of 0.75 or higher considered to be good and 0.75 or lower to be poor-to-moderate [52]. Cronbach’s *α* from 0.7 to 0.8 represents acceptable internal consistency, from 0.8 to 0.9 excellent internal consistency and above 0.9 excellent internal consistency. Additionally, the Standard Error of Measurement (SEM) as well as the Smallest Detectable Difference (SDD) were estimated. The SEM is a metric used to quantify the amount of error in a measurement tool, serving as an indicator of the instrument’s reliability. Analogous to how standard deviation provides a context around a mean value, SEM is utilized to establish a range around an observed value, which it is likely that the actual “true” value exists. An interval of ±1 SEM is associated with a 68% likelihood of encompassing the true value. This probability increases to 95% for ±2 SEM, and to 99% for ±3 SEM. Additionally, the Smallest Detectable Difference (SDD) indicates the minimum amount of change that must be noted to confidently assert that the change is genuine and not merely a result of measurement error inherent to the instrument [52] For the known-group validity, the Mann–Whitney *U* test was used in order to examine the difference of PSQI-GR between asymptomatic and CNSLBP participants. For concurrent validity, the Spearman’s rank correlation coefficient (Spearman’s *r*) was used for the correlation between PSQI-GR, BDI, ISI and SQNRS. Spearman’s correlation coefficient from 0.40–0.59 represents moderate correlation, 0.60–0.79 strong correlation, and 0.80–1 very strong correlation. The significance level was set at *p* = 0.05.

## 3. Results

### 3.1. Participant’s Characteristics

Participant’s demographic and clinical characteristics are outlined in Table 2.

### 3.2. Construct Validity

The mean score for asymptomatic participants was found 5 ± 1.554 and for CNSLBP participants 10.09 ± 5.291 (Table 3). The Mann–Whitney Test showed that there was statistically significant difference between the two groups (*p* = 0.000).

### 3.3. Concurrent Validity

**Figure 1:**
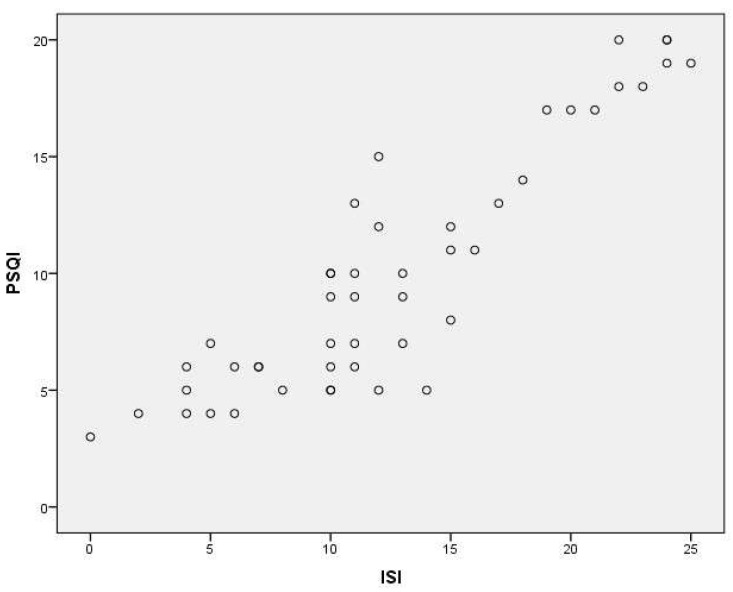
Scatter plot depicting the correlation between PSQI and ISI.

**Figure 2:**
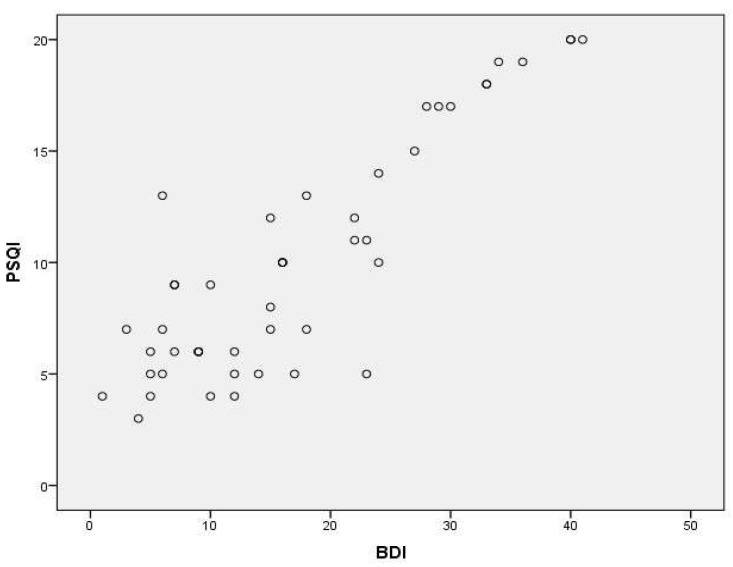
Scatter plot depicting the correlation between PSQI and BDI.

**Figure 3:**
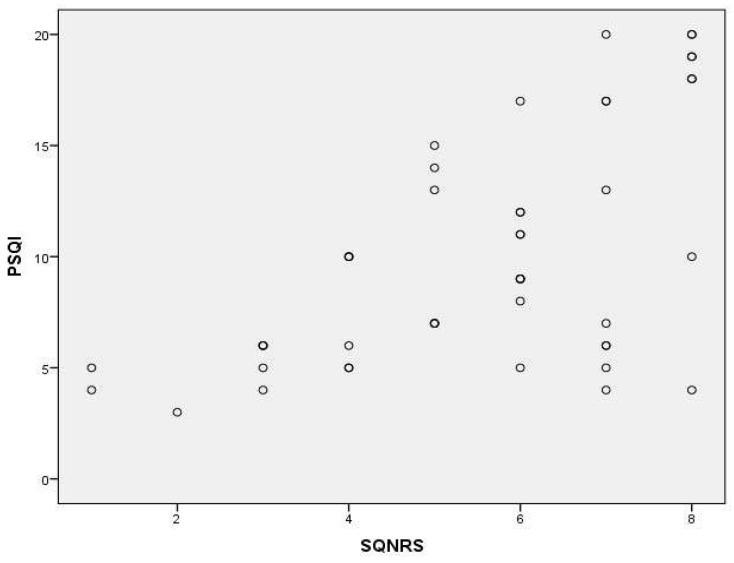
Scatter plot depicting the correlation between PSQI and SQNRS.

According to the results, a high correlation was found between the PSQI and ISI (*r* = 0.860, *p* < 0.01) and between the PSQI and BDI (*r* = 0.786, *p* < 0.01) as well as moderate correlation was found between PSQI and SQNRS (*r* = 0.556, *p* < 0.01) (Figure 1, Figure 2 and Figure 3; Table 4).

### 3.4. Test–Retest Reliability

The test–retest reliability was excellent (ICC = 0.969, SEM = 0.90, SDD = 2.49%), as well as the internal consistency (Cronbach *α* of 0.985 was obtained).

## 4. Discussion

The growing issue of chronic non-specific low back pain (CNSLB) and the necessity for a dependable, validated instrument to evaluate sleep quality in the Greek CNSLBP population prompted the examination of the psychometric characteristics of the Greek Version of the PSQI (PSQI−GR) questionnaire. Originally created in English, this questionnaire has been frequently selected in numerous studies due to its brevity, simplicity, validity, reliability, and ease of use [14]. Furthermore, administrating the PSQI does not require extensive training for researchers or clinicians, making it a practical and widely accepted tool in regular clinical practice for assessing sleep in individuals with chronic non-specific low back pain.

The escalating issue of chronic non-specific low back pain (CNSLBP) and the essential need for a credible, validated instrument to measure sleep quality among the Greek CNSLBP demographic led to the evaluation of the PSQI-GR questionnaire’s psychometric properties. In this study, internal consistency was presented very high (Cronbach’s *α* of 0.985), indicating that the classical seven sleep difficulties components of the questionnaire (sleep quality, sleep latency, sleep duration, habitual sleep efficiency, sleep disturbances, use of sleeping medications, and daytime dysfunction) could effectively evaluate a particular domain of sleep quality. Additionally, the PSQI is advantageous and does not demand extensive training for researchers or healthcare professionals. This makes it a convenient and practical option in daily clinical settings for monitoring sleep quality in individuals dealing with chronic non-specific low back pain. The results of most studies were similar. More specifically, many ICC and Cronbach’s *α* values of PSQI were reported as acceptable values, suggesting it as a reliable tool. Therefore, the ICC value and Cronbach’s *α* value is consistent with those obtained for the PSQI in the most of the different language populations [15,16,17,18,19,20,21,22,23,24,25,26,27,28,29,30,31,32,33,34,35,36,37]. However, studies which employed 1 week time interval, as in the present study, reported the highest test–retest reliability [35]. In contrast, studies that used longer intervals reported lower reliability [15,17,26,28]. An important point is that, the time frame assessed by a measure overlaps significantly between test and retest periods, as with the PSQI, which assesses sleep quality over the past month. Much of the data collected during the 1 week retest will pertain to the same period assessed during the initial test. This overlap could artificially inflate the test–retest reliability coefficient, as the scores are likely to be more similar due to the overlap in the periods being assessed.

For assessing the concurrent validity of the PSQI-GR we used the Greek version of ISI, SQNRS, and BDI. The SQNRS has been qualified as reliable and valid in Greek speakers. The ISI is a reliable and valid tool for insomnia assessment in Greek speakers [15]. Beck Depression Index has been standardized and validated in a sample of Greek speakers as a useful screening measure for depression [53]. The results showed a statistically significant correlation between the PSQI-GR and the ISI, the SQNRS as well as the BDI. The correlation between PSQI-GR and ISI (*r* = 0.860, *p* < 0.001) as well as PSQI and BDI (*r* = 0.786, *p* < 0.001) were the highest compared with the other parameters which were assessed. People with higher scores in BDI present higher scores in PSQI. These findings, possibly related to very strong relation between sleep disturbance and major depression. According to Nutt et al. [54], the relation between the two is so fundamental that some researchers have suggested that a diagnosis of depression in the absence of sleep complaints should be made with caution. People with insomnia, for example, may have a tenfold higher risk of developing depression than people who get a good night’s sleep as well as, among people with depression, 75% have trouble falling asleep or staying asleep. Mood disorders and poor performance status have been systematically associated with altered sleep patterns in patients with chronic non-specific low back pain [55]. Even if correlation between PSQI-GR and SQNRS is lower than those with the previous parameters used, it was statistically significant (*r* = 0.556, *p* < 0.001). Lower correlation between PSQI-GR and SQNRS could be for the reasons below. According to Cappelleri et al. [56], single-item sleep quality assessments, as SQNRS, are practical when measurements are taken at frequent intervals, such as in patient diaries that are completed every day. SQNRS is a self-reported, 11 point numeric rating scale in which respondents report the quality of their sleep over the past 24 h, as an overall rating of sleep. Therefore, it is eligible in evaluations that require a daily record of the quality of sleep, since the respondent is instructed to complete the tool just after waking up, offering the respondent an element with little time burden. Moreover, it may be effective for repeated measures of the same individual, but it is unknown how the specific components taken into consideration might compare between individuals. On the other hand, the PSQI was developed with the understanding that the essential elements that characterize good sleep are mainly subjective and may vary between individuals [57]. Moreover, PSQI as a self-reported, multi-items sleep assessment, addresses specific components of sleep over the past month, that the respondents considered when evaluating the overall quality of their sleep. As a result, the sections of the PSQI provide a tool to compare within and between individuals [56]. Additionally, because the subjective perception of poor sleep quality in people diagnosed with CNSLBP is associated with alterations to different aspects of sleep (e.g., problems falling and staying asleep), tools such as PSQI allow for a more comprehensive assessment of those sleep aspects and could provide valuable information on how poor sleep quality impacts the general health of patients with CNSLBP [57].

Construct validity of the GR-PSQI was also examined. The PSQI global score allowed discriminating between healthy controls and patients. The GR-PSQI demonstrated a satisfactory ability to differentiate patients with chronic non-specific low back pain and asymptomatic people [13,38]. The results of this study are consistent with other studies [2].

The findings of this study hold considerable clinical importance. This is the first time where the PSQI’S validity and reliability have been verified among Greek individuals with chronic non-specific low back pain (CNSLBP). For Greek healthcare professionals and researchers, the PSQI presents as a practical and straightforward instrument for the assessment and tracking of CNSLBP within Greece, offering significant clinical value in addition to its scientific contributions. This research has been instrumental in establishing the PSQI as a widely recognized and reliable clinical tool through investigation of its validity and reliability.

This study has a few limitations that have to be addressed. The sample was not random, thereby, this point could be considered a limitation on the validity of the study. Another limitation was that only some aspects of validity and reliability were assessed (construct and concurrent validity, internal consistency as well as test–retest reliability). Specifically for construct validity, known-groups validity was examined exploring differences solely in global scores but not in factor scores, between groups (asymptomatic participants—CNSLBP participants). Moreover, component-to-component and component-to-total correlations were not examined. Lastly, another limitation is that the choice of 1-week time interval for test–retest, might not be long enough to minimize recall bias therefore, may have inflated the test–retest reliability coefficient. Therefore, further research is needed to evaluate other aspects of validity and reliability (criterion and factorial validity, inter-rater reliability). Moreover, further research in culturally and clinically different low back pain populations is needed to further examine the factor structure of PSQI, as well as to identify the variables contributing to compromised sleep quality in those populations.

## 5. Conclusions

As other CNSLBP populations worldwide, Greek CNSLBP patients may experience severe sleep problems. The existence of psychometrically sound instruments to assess sleep quality, as PSQI, can assist health professionals to effectively address the multifaceted impact of sleep problems on patient’s well-being so as to provide high quality care. Findings from the current study have important implications for the assessment of sleep quality in CNSLBP patients. The PSQI-GR constitutes a valuable, easily applicable, and apprehended tool for use on CNSLBP patients, in both clinical and research environments.

## Figures and Tables

**Table 1:** Reported measurement properties of the PSQI: test–retest reliability and internal consistency.

Study	Language	Sample	ICC	Cronbach’s *α*
Kotronoulas et al. (2011) [15]	Greek	209	0.82	0.76
Curcio et al. (2013) [16]	Italian	50		0.835
Farah et al. (2019) [17]	Malyasian	106	0.58	0.68
Suleiman et al. (2010) [18]	Arabic	50		0.65
Salahuddin et al. (2017) [19]	Ethiopian	311		0.59
Seidi et al. (2019) [20]	Kurdish	230		0.70
Moghaddam et al. (2012) [21]	Persian	125		0.77
Anandakumar et al. (2016) [22]	Sinhala	205		0.85
Dudysová et al. (2017) [23]	Czech	105		0.608
Bertolazi et al. (2011) [24]	Brazilian	104		0.82
Ait-Aoudia et al. (2013) [25]	French	73	0.76	0.72
Sitasuwan et al. (2014) [26]	Thai	138	0.89	0.837
Manzar et al. (2015) [27]	Indian	47		0.736
Sohn et al. (2012) [28]	Korean	394	0.65	0.85
Del Rio João et al. (2017) [29]	Portuguese	347		0.70
Magro et al. (2017) [30]	Maltese	20		0.859
Slochat et al. (2007) [31]	Hebrew	511		0.52
Hashmi et al. (2014) [32]	Pakistan	185		0.56
Hita-Contreras et al. (2014) [33]	Spanish	138		0.805
Escobar-Córdoba et al. (2005) [34]	Columbian			0.78
Takacs et al. (2016) [35]	Serbian	231	0.997	0.791
Setyowati et al. (2020) [36]	Indonesian	528		0.72
Doi et al. (2000) [37]	Japanese	174		0.77

**Table 2:** Participants’ characteristics.

Participant Variable	Asymptomatic	CNSLBP
Participants (n)	73	47
Male (n)	36	22
Female (n)	37	25
Age (mean ± SD) (years)	35.52 ± 12.481	41.3 ± 14.449
Weight (mean ± SD) (kg)	72.92 ± 14.949	76 ± 19.300
Height (mean ± SD) (cm)	1.7197 ± 0.09441	1.7191 ± 0.10172
Intensity of pain (NRS scale)	-	5.57 ± 1.716
Pain duration (months)	-	10.36 ± 10.586

**Table 3:** PSQI means and differences between asymptomatic and CNSLBP participants.

Health Status	n	Min PSQI Score	Max PSQI Score	Mean PSQI Score ± SD	Mean Difference	95% Confidence Interval of the Difference		*p*-Value
						Lower	Upper	
CNSLBP	47	3	20	10.09 ± 5.291	6.523 ± 0.658	5.220	7.827	0.000
Asymptomatic	73	1	9	5 ± 1.554				

Values significantly correlated to the other questionnaires.

**Table 4:** Correlations among PSQI and ISI, BDI and SQNRS.

Sleep Quality Assessment	Spearman Correlation Coefficient	Sig (2-Tailed)	Sample (n)
ISI	0.860	0.000	47
BDI	0.786	0.000	47
SQNRS	0.556	0.000	47

Values significantly correlated to the other questionnaires.

## Data Availability

Data are contained within the article.
